# Comparative Physiology of Energy Metabolism: Fishing for Endocrine Signals in the Early Vertebrate Pool

**DOI:** 10.3389/fendo.2017.00036

**Published:** 2017-03-02

**Authors:** Iris van de Pol, Gert Flik, Marnix Gorissen

**Affiliations:** ^1^Department of Animal Ecology and Physiology, Institute for Water and Wetland Research, Radboud University, Nijmegen, Netherlands

**Keywords:** leptin, insulin, gills, metabolism, aerobic scope, oxygen, teleost, fish

## Abstract

Energy is the common currency of life. To guarantee a homeostatic supply of energy, multiple neuro-endocrine systems have evolved in vertebrates; systems that regulate food intake, metabolism, and distribution of energy. Even subtle (lasting) dysregulation of the delicate balance of energy intake and expenditure may result in severe pathologies. Feeding-related pathologies have fueled research on mammals, including of course the human species. The mechanisms regulating food intake and body mass are well-characterized in these vertebrates. The majority of animal life is ectothermic, only birds and mammals are endotherms. What can we learn from a (comparative) study on energy homeostasis in teleostean fishes, ectotherms, with a very different energy budget and expenditure? We present several adaptation strategies in fish. In recent years, the components that regulate food intake in fishes have been identified. Although there is homology of the major genetic machinery with mammals (i.e., there is a vertebrate blueprint), in many cases this does not imply analogy. Although both mammals and fish must gain their energy from food, the expenditure of the energy obtained is different. Mammals need to spend vast amounts of energy to maintain body temperature; fishes seem to utilize a broader metabolic range to their advantage. In this review, we briefly discuss ecto- and endothermy and their consequences for energy balance. Next, we argue that the evolution of endothermy and its (dis-)advantages may explain very different strategies in endocrine regulation of energy homeostasis among vertebrates. We follow a comparative and evolutionary line of thought: we discuss similarities and differences between fish and mammals. Moreover, given the extraordinary radiation of teleostean fishes (with an estimated number of 33,400 contemporary species, or over 50% of vertebrate life forms), we also compare strategies in energy homeostasis between teleostean species. We present recent developments in the field of (neuro)endocrine regulation of energy balance in teleosts, with a focus on leptin.

## Introduction

Among contemporary vertebrate species, none are as abundant as the teleostean fish. With an estimated number of 33,400 species, fish comprise roughly half of all vertebrates (1). The earliest vertebrates originated approximately 530 million years ago (Mya) in the Panthalassan, Paleo-Thetys, and Iapetus oceans (2, 3). The rise of primitive fishes in the Ordovician was followed by an unparalleled radiation of the aquatic vertebrates in the Devonian period (the “Age of fish”), and next, ~380 Mya, after the water–land transition, terrestrial tetrapods radiated (4, 5).

Before the teleost–tetrapod split, at least two whole (or large-scale) genome duplication (WGD) events occurred (6). These duplication events have been paramount in expanding the functional gene repertoire of all vertebrates and facilitated functional divergence of genes. The “Ohno-mechanism” (2) states that of a pair of duplicated genes, one can retain its original function while the other one is silenced to become a pseudogene (is no longer expressed) or acquire a new function, and this is called neo- or sub-functionalization (7). These phenomena may also explain why fish lineages escaped from five major mass extinctions (and many smaller ones) that raged on earth and challenged all life forms (8).

Within the class of the Actinopterygii (ray-finned fishes), a third genome duplication occurred around 350 Mya (9); in cyprinid and salmonid lineages, even a fourth major duplication occurred (10, 11). An attractive hypothesis is that the phylogenetic timing of these events suggests that fish-specific genome duplications accommodated the extent of radiation and phenotypic diversification seen in the teleostean lineage (12). However, ecophysiological factors appear to be primary drivers of the rate of salmonid diversification, not the salmonid WGD *per se* (13). Indeed, a more extensive study shows that there is no direct association between the teleostean WGD and the *rate* of diversification in lineages (14). Whatever mechanism prevails, of all extant actinopterygian fishes (except for ~44 basal non-teleostean species), the teleosts (the largest group of bony fishes) comprise, with about 96%, the majority of all fishes (9, 12, 15, 16).

Modern bony fish are found in virtually all aquatic niches imaginable (17) and even in terrestrial environments (lungfishes, mudskippers) and display amazing adaptations in energy homeostasis. Fish have adapted to extremely challenging environments offering them either high tolerance or strong acclimation capacity to unfavorable conditions (18). Indeed, as energy homeostasis requires regulation of the balance between anabolism and catabolism, between energy intake and expenditure, we find adaptations in both. Energy intake essentially equals food intake, expenditure concerns adenosine trisphosphate (ATP)-consuming processes and thermogenesis. Breakdown of energy carriers (sugars, fatty acids, and protein) will free energy that is then temporarily stored in ATP. This molecule is, therefore, called the “currency of energy” (19). Under anaerobic conditions, in a fermentation pathway, glucose breakdown to pyruvate yields only two ATP’s, while under aerobic conditions and operationalizing the citric acid cycle a total of up to 38 ATP’s are formed per glucose molecule.

In the first part of this review, we discuss key physiological aspects of energy metabolism that determine how energy is budgeted: metabolic strategies, thermal physiology, and aerobic scope are addressed with a special attention to the earliest vertebrates, fish. We (non-exhaustively) look at changes in the regulation of the metabolic demand depending on an organism’s thermal physiology. In the second part, we discuss the consequences of these different physiologies for the endocrinology of energy metabolism, with a focus on insulin and, mainly, leptin. These hormones are key in manipulating energy stores, i.e., the regulation of energy intake and expenditure, on the long term.

## Living in an Aquatic Niche

The inefficiency of metabolism under hypoxia (a regular phenomenon in water bodies) or anoxia must have favored adaptations toward optimization of branchial oxygen uptake mechanisms, oxygen carrying capacity by adjustment of hematocrit, hemoglobin content or oxygen affinity, or facilitating anaerobic metabolism. Fish, gill-bearing vertebrates, threatened by hypoxia invest in adaptations that boil down to adjustments in oxygen provision and rely secondarily on modifications of metabolic pathways.

When oxygen levels become limiting or 0, fish living in such niches exhibit strong metabolic suppression and use fermentation pathways for ATP production as escape; goldfish (*Carassius auratus*), bitterling (*Rhodeus amarus*), and crucian carp (*Carassius carassius*) avoid lactate accumulation by pyruvate dehydrogenase-mediated production (in their muscle compartment) of acetaldehyde and ethanol, which is excreted *via* the gills. By doing so, potentially lethal lactic acidosis is avoided (20, 21).

Antarctic icefishes (with many representatives in the families of *Nototheniidae* and *Channichthyidae*) live at ambient seawater (SW) temperatures of −1.9°C and below [having evolved antifreeze proteins that prevent ice crystals in their bodily fluids from growing and by doing so, prevent cryodamage (22)] in normoxic seawaters. Although more oxygen dissolves in colder water, the bioavailability of oxygen is much lower (23). This is due to lower O_2_-diffusion rates in the cold, reducing an organism’s capacity to take up oxygen and ensure an adequate oxygen supply. Icefishes have lost hemoglobin (Hb) (and lack red blood cells) and often also myoglobin (Mb). This remarkable feature is not, as was widely assumed, an adaptation to prevent their blood from becoming too viscous. In fact, in terms of energetic cost, it would be a disadvantageous adaptation, as icefishes pump a far greater blood volume per time unit than “red-blooded” teleosts of equal body mass (24). Instead, the loss of Hb and Mb includes the loss of a primitive function of these proteins: the oxygenation of NO to ${\text{NO}}_{3}^{-}$ (25). Indeed, icefishes have high concentrations of circulating NO, which stimulates vasodilation, angiogenesis, and mitochondrial biogenesis. Hence, elevated NO levels might have been the evolutionary driver of unique adaptations in the oxygen delivery system of icefishes [reviewed in Ref. (24)]. The very-low aerobic metabolism of these carnivores depends merely on oxygen diffusion over an enlarged gill surface from water to blood and from blood to tissues from a large vessel bed; a large heart, with cardiocytes and with an extreme mitochondrial density, pumps the blood through the vessel bed that is expanded to compensate for the limited oxygen carrying capacity of the plasma (26).

The scaleless carp (*Gymnocypris przewalskii*) meets extreme conditions, including chronic mild hypoxia, in Lake Qinghai [6 mg O_2_ L^−1^; 9–13 ppt salinity; pH ~9.3 (27)] on the Tibetan Plateau at 3,200 m. When challenged with extreme hypoxia (0.3 mg O_2_ L^−1^), its gills are remodeled by lamellar expansion in a matter of hours to increase (diffusional) oxygen uptake (27). The inherent consequences for hydromineral disturbances are counteracted by changes in ion channel and aquaporin expression in osmoregulatory organs (28).

The crucian carp survives over six dark winter months of anoxia by strong metabolic suppression. The mechanisms of hypoxia/anoxia-tolerance of this fish are only partly understood; preferentially, the fish stores energy in glycogen, and the glycogen volume determines the duration of hypoxia it can survive. Preceding the switch to anaerobic metabolism (see above), the crucian carp remodels its gills in response to the imminent anoxic conditions. An extensive gill remodeling takes place under control of hypoxia-inducible factor 1α (HIF-1α) (29): the gas exchange surface increases due to a reduction of filamental epithelial thickness resulting in a further protrusion of lamellar epithelium. This comes with a cost as energy consuming osmoregulatory adjustments are required to compensate diffusional flows of Na^+^ and Cl^−^ (reminiscent of the situation in the scaleless carp) over the expanded gill surface. The investment in an expansion of the machinery to obtain oxygen has high priority to keep on fueling aerobic metabolism. Next, anaerobic metabolism offers an escape when oxygen diffusion becomes limiting to fuel the citric acid cycle.

The Magadi tilapia (*Alcolapia grahami*) from Lake Magadi in Kenya is described as the “hottest fish on earth”: it thrives in highly alkaline (pH 9.8), hypersaline (880 mOsmol kg^−1^) waters with temperatures over 40°C (critical temperature: 45.6°C, i.e., above this temperature loss of equilibrium and often death occurs). The concentration of oxygen in SW (at 35‰ salinity, comparable to the water in which this fish lives and 101.1 kPa pressure^1^) will drop from 7.2 mg L^−1^ at 20°C to 5.3 mg L^−1^ at 40°C, and, thus, although a large water body in principle provides an infinite oxygen source for the fish, its uptake mechanisms need adjustment to compensate for this roughly 30% drop in water oxygen content. Indeed, upon comparison with the same species kept at lower temperatures, compensation was seen in a very high mass-specific gill area to facilitate oxygen uptake, a high metabolic rate (of which an estimated 50% seems needed for acid–base regulation), and a high mitochondrial respiration rate. Moreover, because of the basic environment and its consequences for ammonium excretion, this fish is 100% ureotelic, i.e., it does not produce ammonium but the energetically costlier urea as waste (30). The rates of O_2_ consumption and swimming performance at 39°C in laboratory setting indicate that this tilapia exhibits the greatest metabolic performance recorded in any fish, in the basal metabolic rate (BMR) range of a similar sized shrew (31).

## Physiology

### Metabolic Rates

Metabolism (*sensu lato*) can be defined as “the complex of physical and chemical processes involved in the maintenance of life” (32). Metabolic rate is a measure for the amount of energy used per unit of time by an organism, generally assessed as rate of oxygen consumed per hour [e.g., O_2_ consumption in millimoles per hour (33, 34)]. In fish and other bradymetabolic animals (see Data Sheet S1 in Supplementary Material), and these are mostly ectotherms, the standard metabolic rate (SMR) and maximum metabolic rate (MMR) are generally used as ratios to define the “scope for activity” (35). SMR refers to the minimal rate of energy expenditure in an organism at rest. Basically, this equals the BMR in the tachymetabolic birds and mammals. However, SMR can be influenced by ambient temperature (36). MMR indicates the highest possible rate of energy expenditure, e.g., during sustained and aerobic maximal activity. In between these states, the routine metabolism is defined as energy expenditure during spontaneous activity in common life processes (35). Note that only the aerobic metabolism is taken into account when metabolic rate is measured *via* oxygen consumption.

### Ectothermy Meets Endothermy

The majority of animal life forms are ectothermic (see Data Sheet S1 in Supplementary Material for discussion on terminology). The environment determines the body temperature of ectotherms for the major part and, with that, the pace of biochemical reactions and rates of physiological processes. If we go back to the Magadi tilapia, we have seen, to the best of our knowledge, the upper limit of metabolic performance of an aquatic ectotherm. Note that the maximum performance of this fish comes to 35% of that of a similar sized mammal (31). After the water–land transition of vertebrates (~390–360 Mya) and the evolution of endothermy, new niches of the terrestrial environment became available. True endothermy (by this we do not mean regional endothermy, as we will discuss below) evolved independently in two different clades: (i) the diapsid clade that gave rise to extant birds and (ii) the synapsid clade that gave rise to the contemporary mammals (37). Animals that invest in endothermy (birds, mammals) can stay active independent of meteorological conditions, but do so at phenomenal cost [i.e., it requires lots of fuel (38)].

One could argue that ectotherms function at the mercy of ambient temperature; however, they are not passive and have the potential to regulate their body temperature. By shifting thermal preference and actively migrating toward warmer or colder environments, ectotherms are capable of sophisticated and adaptive behavioral thermoregulation (39, 40). To cope with challenges like infections or stressors (41–43), some fish migrate to warmer water, i.e., behavioral fever, a process that should be regarded as an intrinsic feature of ectothermic physiology.

Indeed, ectothermy comes with many benefits: it is energetically more economical, since the energy demand per unit mass, compared to an endothermic animal of the same size, is four to five times lower (44). Therefore, ectotherms require less time for foraging, and by doing so reduce their energy demand even more as a result of less locomotor activity. Moreover, as ectotherms need not invest in maintenance of a relatively high and constant body temperature, they can allocate more energy to growth and reproduction (44).

Here, we seem to reach a paradox, as endothermy also has advantages over ectothermy. Besides relative independence from ambient temperatures, energy metabolism is significantly enhanced in a warm body, which allows for high and fast muscular activity, fast metabolism, and growth during circadian and seasonal fluctuations. Notably, locomotion requires much more energy in terrestrial than in aqueous environments [the energetic cost for transport is about 10 times higher in terrestrial, running or walking, vertebrates than in equal-sized swimmers (45)], which might have been an important determinant in the evolution of endothermy. In addition, endothermy provides a stable thermal environment, securing optimal enzyme activities (46) and facilitating the ultimate parental care seen in mammals (47, 48).

What drove the evolution of endothermy? Three major hypotheses addressed this question. Crompton et al. (49) proposed that endothermy evolved in a two-step process: first, a more or less constant body temperature was acquired; next, the body temperature and metabolic rate increased. McNab (50) argued that early reptiles with a large body mass were *de facto* inertial homeotherms. Following an evolution of fur and decreasing body size, with a modest increase in mass-specific metabolic rate, inertial homeothermy became true endothermy. The most widely accepted theory, however, was put forward by Bennett and Ruben (38), who argued that higher body temperatures and endothermy have evolved secondarily to the selection on enhanced maximal aerobic capacity. This is known as the aerobic scope hypothesis. The evolution of endothermy is maybe the single most debated topic in comparative biology. The proposed hypotheses are not mutually exclusive. For this review, we focus on the aerobic scope hypothesis, as it conveniently joins aspects of metabolic demand and energy balance.

Bennett and Ruben (38) showed that the ratio between resting and maximal metabolic rates in vertebrate ectotherms and endotherms is roughly the same [on average 10 (51)], although the resting metabolic rate in endotherms is around 10 times higher than in ectotherms of similar body mass, i.e., the factorial aerobic scope is comparable for ectotherms and endotherms, but the absolute aerobic scope (the difference between resting and maximal metabolic rates) is much greater in endotherms. As a result, endotherms have, in general, more energy available for processes other than resting metabolism. The aerobic scope hypothesis holds aerobic metabolism at its center, which is important in sustained muscular activity. Still, a reptile can outrun a mammal, achieving great muscular power through anaerobic metabolism (52). This burst activity is time limited, as lactic acid builds up; some lizards and amphibians are highly tolerant to these metabolic waste products (53, 54).

Clarke and Pörtner (37) modified the original aerobic scope hypothesis: they regard a higher body temperature as the mechanism by which a greater aerobic scope was achieved, rather than as a consequence. They point out that an endotherm is not merely a warm ectotherm, since several processes are linked to the evolution of endothermy, such as the modification of mitochondrial membranes and heat retention (insulation by feathers, hair, and fat) mechanisms. In their view, the evolution of endothermy is considered a gradual process in which the driving force was selection for increased aerobic scope (37).

The magnitude of aerobic scope is greatly influenced by temperature. An optimal aerobic scope, associated with a species’ thermal niche specialization, is of vital importance for their performance and fitness [reviewed by Pörtner et al. (55)]. The first indication for thermal intolerance in ectotherms is a decreased aerobic scope due to a mismatch in oxygen supply and demand in tissues (56). Even before critical temperatures (here defined by the switch from aerobic to anaerobic metabolism) are reached, failure of circulatory and ventilatory systems occurs, which affects all higher functions (e.g., locomotor activity, behavior, growth, and reproduction) (57). Hence, even slight decreases in aerobic scope can result in lowered performance and enhanced mortality. The mechanisms that are crucial for aerobic scope and related to thermal intolerance are best studied in cold-adapted eurytherm and stenotherm fishes. These special adaptations allow them to withstand seasonal and permanent cold, respectively (58).

Eurythermal fishes are those that tolerate a wider thermal range than stenothermal fishes, and this relates to their ability to increase mitochondrial density or capacity in the cold to prevent hypoxia in tissues (58, 59). This is energetically costly, since they need to upregulate their SMR for mitochondrial maintenance. Stenotherm fishes have a permanently low SMR, associated with specialized cold-adapted mitochondria. These are found in extremely high densities in aerobic tissues, but do not increase overall aerobic capacity (60). Sidell (61) explained that the high density of enlarged mitochondria, seen in the icefishes lacking Hb and Mb, forms an interwoven membrane network, acting as a “lipid highway” for oxygen delivery. Selection for energy savings may have narrowed the thermal window for stenotherm fishes to survive with minimal aerobic capacity and energy expenditure, but allows them to preserve metabolic energy for processes like growth and reproduction, which are temporarily suspended in cold-acclimatized eurytherm fishes (55, 58).

Where is the heat coming from in an endotherm? During generation of ATP in the mitochondria, highly energetic electrons pass through the electron transfer chain and protons are pumped from the mitochondrial matrix into the intramembranous space. Following build-up of that gradient, protons flow back over the mitochondrial inner membrane and drive an H^+^-ATP synthase. As membranes are somewhat leaky for protons, some protons passively diffuse over the membrane and, without being used for ATP synthesis, emit energy as heat. Controlled uncoupling *via* the mitochondrial uncoupling proteins (UCPs), of the proton flux from ATP generation was thought to be the main source of heat in endotherms (62). However, more recent research shows that UCPs are not the exclusive actors in proton conductance, as the observed proton leak contributes proportionally and equally to the SMR in ectotherms and endotherms (63).

In mammals, five UCPs are present. In mammals UCP1 is responsible for non-shivering thermogenesis, whereas UCP2 and UCP3 are thought to be involved in maintaining the resting metabolism. In fish, we find orthologs of UCP1, UCP2, and UCP3, illustrating that the presence of UCPs is not restricted to endothermic animals (64, 65). In common carp (*Cyprinus carpio*), hepatic *ucp1* transcript abundance was significantly downregulated in response to cold (opposing the function of UCP1 in mammals). Note that the fish liver is a primary storage site of energy. In red muscles, *ucp3* is predominantly expressed and increases up to fivefold in response to fasting (65). As fasting leads to high lipid oxidation and reactive oxygen species, UCP3 is suggested to protect mitochondria and cells from damage *via* mild uncoupling activity or fatty acid anion export (66–68). The omnipresent UCP2 may play a similar scavenging role. Certainly, UCPs are evolutionary much older than originally thought; they were already present in fish ~420 Mya (65).

Although most fishes are strictly ectothermic, there are (of course) also fish that attain a regional endothermy in important organs and tissues, such as muscles, eyes, visceral organs, and the brain (69). We follow the nomenclature proposed by Clarke and Pörtner (37) (see Data Sheet S1 in Supplementary Material), and name this process heterothermy. An exceptional group of teleosts capable of heterothermy are the *Thunnini* (tuna).

Tuna are obligate ram ventilators and they are not sufficiently buoyant, which means they have to swim continuously to maintain a constant water flow over their gills (to fuel their relatively high metabolic activity) and to prevent sinking (70). Tuna have a specialized red muscle (RM) that is constantly metabolically active to power this so-called cruise swimming. Since the byproduct of all metabolic processes is heat, tunas have developed a way to retain the heat that is generated by RM and use it to elevate their body temperature regionally (69).

In tuna, the circulation to and from RM is structurally arranged in so-called *retia mirabilia* (literally: wonderful nets) (71). In these parallel to one another organized vascular bundles with very thin vessel walls, arterial and venous vessels lay side by side with the blood flowing in opposite directions, so that they function as countercurrent heat exchangers. Hence, a stable thermal gradient is established, which warms the cool arterial blood coming from the gills. The heat conserved is used to warm other body parts (72). When tuna dive during predation from pelagic zones with high temperature (e.g., 20°C) to more benthic zones where temperatures may drop to around 4°C, the biochemistry of brains, eyes, and swimming muscles is guaranteed of a rather constant temperature.

Considering that the SMR of tuna is 2–10 times higher than in most other fish species of comparable size and activity (69), this demonstrates that metabolism, and more specifically metabolic rate, is at the basis of thermal regulation in both ecto- and endotherms. The production of heat and having heat-retaining mechanisms are equally important. In general, maintaining the temperature of (part of) the body above the ambient temperature, is energetically costlier than keeping the body at lower temperatures, due to higher standard or basal metabolic rates. However, it also results in enhanced ATP production, which in turn allows for higher locomotor activity, growth rate and, completing the circle, metabolic rate. To understand causality, one needs to take a closer look at the regulation of energy intake and expenditure, and a comparative endocrine view may facilitate this, as we will do in the next section. Yet, it is clear why temperature is regarded as the abiotic master factor (73).

## Endocrinology

### Regulation of Energy Balance

Metabolism, thermoregulation, and aerobic performance are intertwined and have one nexus in common: they are all dependent on food intake, food being the source of chemical energy for these related processes. In general, and despite mismatches in energy intake and expenditure on the short term, on the long term, they are carefully balanced and regulated by several endocrine systems, which together guarantee energy homeostasis (74).

A plethora of research on mammals is available in this field, not in the least to gain a better understanding of disturbances in energy homeostasis, which are pivotal for the current obesity pandemic (75, 76). In a healthy animal, the control system that matches energy intake to energy expenditure is remarkably accurate. A striking example is put forward by Seeley and Woods (75), who calculated that it takes only a mismatch of 46 kJ per day (0.55% of the daily energy intake!) for an adult human male to gain a pound in a year. Such tight regulation of energy balance can only be achieved by an accurate integration of signal molecules from stored and currently available fuel, in accordance with internal set points reflecting energy availability.

Where these signals come from and how they contribute to the regulation of food intake is of ongoing research interest. It has long been recognized that specific hypothalamic nuclei are crucial in monitoring energy balance (77), and these seem to be mainly tuned by signals regarding the storage of fat (i.e., lipostatic regulation). Another theory about hypothalamic tuning of energy balance through signaling of carbohydrate storage [i.e., glucostatic regulation (78)] has largely been abandoned in the past decades, as it has become clear that neurons are well protected from fluctuating glucose levels and usually feeding takes place when plasma glucose levels are within normal physiological values (75).

The link between body-fat stores (adiposity) and food intake was formalized by Kennedy (79), when he postulated that signals distributed in proportion to the total amount of body fat influence the control of food intake by the brain. This lipostatic regulation is based on a negative feedback system involving communication on the total amount of fat by adiposity signals and the central nervous system (CNS) (80).

Since adipose tissue is poorly innervated by the peripheral nervous system, research has been directed mainly to humoral signals. Adiposity signals (see below) should fulfill three criteria, *viz*.,: they should circulate in proportion to the total body fat, they should reach specific nuclei within the CNS [i.e., cross the blood–brain barrier (BBB)], and they should produce predictable changes in energy balance by altering food intake and energy expenditure dose-dependently (74).

### Insulin and Leptin: Adiposity Signals in Mammals

In the 1980s, ample evidence identified insulin as a major adiposity signal (81, 82). Porte and Woods (81) showed that intracerebroventricular (icv) infusion of insulin in baboons induced drastic weight loss and decreased food intake dose-dependently. In additional studies, insulin receptors were found in key brain areas involved in control of food intake, including the hypothalamic arcuate nucleus (ARC, see below) (83, 84). Insulin is shuttled over the BBB *via* receptor-mediated transport (85). Insulin, produced by pancreatic β-cells, is best known for its involvement in carbohydrate metabolism; in response to high plasma glucose levels, insulin enhances the uptake of glucose by adipocytes, liver, and skeletal muscle cells, storing energy as glycogen or triglycerides (86). Insulin circulates in proportion to the amount of adipose tissue, although its levels fluctuate greatly when food is ingested and absorbed, in accordance with its hypoglycemic actions. However, baseline insulin levels and the magnitude of the fluctuations following food intake are directly proportional to body adiposity (87).

A second adiposity signal, leptin, a type-I α-helical cytokine, is encoded by the *obese* (*ob*) gene and was discovered in mice in 1994 (88) and has gained unparalleled momentum in research on energy homeostasis. It is a potent anorexigenic hormone produced in peripheral white fat and meets the aforementioned criteria for an adiposity signal. In addition, receptors for leptin are present in brain areas involved in the regulation of food intake, especially in the ARC (75).

Two subpopulations of neurons in the ARC integrate peripheral signals regarding energy homeostasis: the “anabolic” population expresses neuropeptide Y and agouti-related peptide, is orexigenic, and inhibited by leptin (89), whereas the “catabolic” population expresses pro-opiomelanocortin and cocaine and amphetamine regulated transcript, is anorexigenic, and stimulated by leptin (90). In line with this model, leptin administration in rodents was shown to decrease body mass dose dependently (91, 92). During fasting, plasma leptin levels fall dramatically and over prolonged periods of food deprivation, changes in the activities of the gonadal, adrenal, and thyroid axes are observed (93). Postprandially, leptin does not increase significantly, nor does it lead to termination of a meal by itself, indicating that leptin is largely involved in long-term regulation of feeding behavior and energy balance (94).

Leptin administration is an effective treatment for obesity in mice and humans with genetic leptin deficiency (95–97). However, in most cases of obesity, circulating leptin levels are already high and leptin therapy is not effective. Indeed, leptin resistance and the consequent lack of anorexic signaling in the ARC is commonly associated with obesity (98). Interestingly, leptin resistance in the ARC does not cause obesity, but it contributes to its persistence, as it develops secondarily after adiposity and body mass increase (99). More recent research revealed that vagal afferent neurons (VAN), apart from their role in meal termination *via* short-term gut–brain signaling, are involved in the long-term regulation of food intake (100–102). The authors showed that leptin resistance developed in VAN, before hypothalamic leptin signaling became disturbed (100). Moreover, when the leptin receptor is knocked out in VAN specifically, mice displayed increased food intake, body mass, and adiposity, indicating that the absence of leptin signaling in VAN is an important factor in the onset of hyperphagia and obesity (101).

Both adiposity signals (insulin and leptin) exert their function centrally through signaling *via* the ARC neurons, which results in reduced food intake and increased energy expenditure (103, 104). Arguably, leptin, for a while thought to be the panacea to beat obesity (105), received major attention in obesity research, whereas the role of insulin was less appreciated. This might be due to the opposing actions of insulin in peripheral tissues and the CNS; its hypoglycemic and anabolic function (energy storage) peripherally; and its catabolic effect (steering energy expenditure) in the hypothalamus (106). In the periphery, however, obesity eventually leads to inflammation of adipose tissue, and the following interplay between leptin signaling and the inflammatory cytokines secreted by macrophages seems to contribute to insulin resistance (107).

The complementary, and sometimes redundant, roles of leptin and insulin in the central regulation of energy homeostasis are best illustrated in a condition known as diabetic hyperphagia. When pancreatic β-cells become non-functional, complete depletion of insulin leads to strong hyperglycemia and an inability to store energy peripherally, with considerable weight loss as a result. However, without the catabolic insulin action in the hypothalamus and the co-occurring decrease in plasma leptin levels, increased compensatory food intake is initiated, counteracting energy wasting and preventing more rapid weight loss (106). Administration of either leptin or insulin to the CNS in rats with streptozotocin-induced diabetes blocks this compensatory hyperphagia almost completely (108, 109).

**Figure 1:**
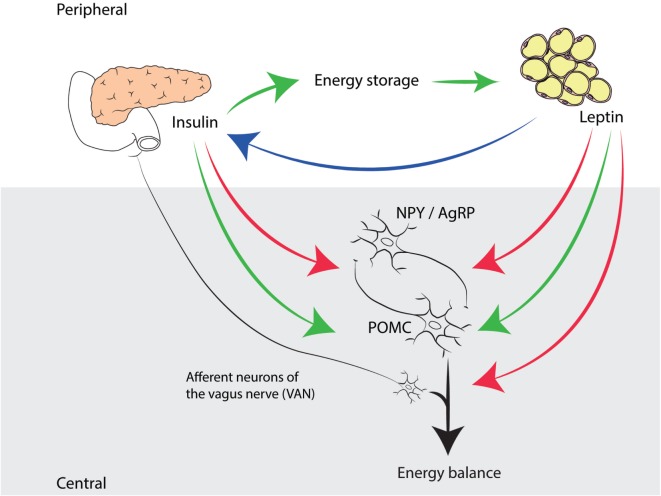
**Complementary roles of insulin and leptin in energy homeostasis in mammals**. Insulin, produced by pancreatic β-cells, has an anabolic effect in peripheral tissues by promoting glucose storage, which has a positive effect on body weight and adipose tissue. Leptin, produced by adipocytes, signals to the hypothalamic arcuate nucleus, stimulating POMC expressing neurons (enhancing energy expenditure) and inhibiting NPY/AgRP expressing neurons (promoting food intake). In addition, leptin signals to VAN, which, according to recent insights, directly influences energy balance. Insulin also has a catabolic effect on the CNS, by exerting the same functions as leptin in the ARC. Note that insulin levels are proportional to adiposity (indicated by a blue arrow).

In fact, one can state that leptin and insulin are equally important adiposity signals involved in a negative feedback loop, as has been shown in many studies before (Figure 1) (75, 103, 110, 111). We focus on leptin mainly in this review, because leptin received the lion’s share of attention in recent research; however, we recognize the significance of the undervalued role of insulin. As Seeley and Woods (75) point out, insulin provides the brain with information not only about fat storage (long-term energy) but also about glucose availability (short-term energy). From this perspective, although indirectly, glucostatic regulation of food intake is indeed taking place. Thus, insulin signaling to the brain could provide the link that integrates glucostatic and lipostatic peripheral signals, allowing for a precise monitoring and accurate regulation of energy balance.

### Insulin and Leptin: Adiposity Signals in Fish?

The insulin signaling pathway and its role in energy metabolism is evolutionary conserved and serves fundamentally the same physiological functions from invertebrates to mammals; it is found in phylogenetically distant invertebrate species, such as *Caenorhabditis elegans* and *Drosophila melanogaster*, where it is involved in energy storage (106, 112–114). Interestingly, these animals express homologs of key players in the insulin signal transduction pathway, but do not seem to actively regulate carbohydrate fluxes. They do regulate fat stores: insulin appears to limit energy storage in *C. elegans* (mutants for insulin signal transduction had increased fat deposits), which indicates a catabolic function of this protein in early evolution (106).

In elasmobranchs (mostly carnivores), protein and lipid metabolism are the primary energy sources (115), and although infusion of mammalian insulin resulted in severe prolonged hypoglycemia in spiny dogfish (*Squalus acanthias*) (over 15 times lower than time-zero levels), these fish did not exhibit any symptoms of illness (116). Apparently, strict glucose regulation is not as vital in these animals as it is in later vertebrates. The structure of insulin and its receptor is also highly conserved among vertebrates (117). Fish insulins, as their mammalian orthologs, consist of an α- and β-chain, linked by disulfide bridges (118); when we align the zebrafish an human insulin genes, we find a sequence of 60%.

As in mammals, it is important to distinguish between peripheral and central functions of insulin. The role of insulin in the periphery as an anabolic hormone has gained more attention, as it was long, but erroneously thought that fish were glucose intolerant [reviewed in Ref. (117)]. He argues that metabolic rate is a factor commonly overlooked in the studies performed on glucose clearance in fish. Acknowledging the fact that the metabolic rate of fish is about 10 times lower than in mammals, it may not come as a surprise that glucose turnover is rather slow (119). For several fish, it was shown that amino acids are a more potent stimulator of insulin secretion than glucose (120, 121), and fish are in general much slower in clearing a glucose load, compared to mammals (117). Notwithstanding, hyperglycemia does induce hyperinsulinemia *in vivo* (122–124). Significant differences between species exist: in the herbivorous carp glucose is more potent in stimulating insulin secretion than it is in carnivorous species, such as trout (*Oncorhynchus mykiss*) and sea bream (*Sparus aurata*) (125).

Contrasting results have been found in studies that address the central role of insulin. Fish brains are likely insulin-sensitive organs, as they contain insulin receptors (126–128). In channel catfish (*Ictalurus punctatus*), 24 h of icv injection of insulin appeared to have no effect on food intake (128). In contrast, icv administration of insulin in rainbow trout elicited a reduction of food intake after 26 h (129). Moreover, it has been shown in rainbow trout that insulin levels drop dramatically after 6 weeks of fasting, a response also seen in mammals (130). These conflicting outcomes could be the result of the longer administration time in the trout study or, more likely, central insulin effects could be species dependent. Progressive insight suggests that fish, like mammals, have specialized neurons involved in glucose sensing, which modulate the hypothalamic neurocircuitry controlling food intake (131).

Whether insulin has similar functions in fish as in mammals in communicating adiposity status to the CNS is not evident. In studies on rainbow trout, insulin stimulates lipogenesis in the liver and, more recently, in white mesenteric adipose tissue (132, 133). Traditionally, researchers considered the fish liver to be the main lipogenic tissue (134). However, Polakof et al. (133) showed that in rainbow trout fed a carbohydrate-rich diet and provided with insulin, lipogenesis in adipose tissue was significantly enhanced. The researchers hypothesized that the increased lipogenic capacity helps to regulate glycemia when excess carbohydrates need to be processed. In addition, these results are indicative for the utilization of lipids, stored in white adipose tissue, as an energy source. In juvenile chinook salmon, circulating insulin levels increased with a high-fat diet, and body adiposity clearly reduced food intake compared to fish that were fed a low-fat diet (135). However, no direct association between insulin levels and adiposity was found, but insulin levels correlated with dietary fat content.

More than a decade after the breakthrough identification of the mammalian *ob* gene and its protein product leptin (1994), the first non-mammalian ortholog was characterized in a teleostean fish [common carp (136, 137)] and many other fish species followed, including zebrafish (*Danio rerio*) (138), Atlantic salmon (*Salmo salar*) (139), and tiger pufferfish (*Takifugu rubripes*) (140). More recently, the first non-vertebrate functional leptin homolog was identified in *Drosophila* (141). The *Drosophila* cytokine Unpaired 2 has structural and functional similarities with mammalian leptin, is secreted by the fat body and signals *via* a similar intracellular messenger signaling pathway to reduce growth and alter energy metabolism. The canonical paper by Zhang et al. (88) addressed the *ob* gene as “evolutionary conserved” after demonstrating its presence in evolutionary distant species like *Drosophila* and eel (*Anguilla rostrata* LeSueur). However, once fish leptin was cloned and sequenced in 2004 (Huising et al.; accession numbers AJ868357 and AJ868356), the primary sequence identity between fish leptins and mammalian leptins turned out to be less than 25% (136), at variance with the earlier proposed “evolutionary conservation.” However, conserved gene structure, phylogenetic analyses, the conservation of essential cysteine residues, and tertiary structure, as well as synteny, firmly demonstrated true orthology of teleostean leptins (136, 138, 140). This indicates that if one wants to study the original function(s) of leptin(s), fish should be addressed.

The organization of hypothalamic nuclei involved in energy metabolism and food intake is conserved throughout the vertebrate lineage (142, 143), yet reports on leptin’s physiological role(s) have not been consistent for different fish species, which may not be surprising considering the vast number of species and niche adaptations. Most studies, however, recognize the organs and tissues with a high-fat content (liver or muscle) as the main expression sites for leptin in fish, not adipose tissue like in mammals (136, 138–140, 144, 145). In contrast with mammals, the liver is one of the major energy depots (glycogen and fat) in fish, together with muscle tissue and mesenteric fat (146). Moreover, lipid catabolism is the main source of energy in many fish species (147). Next to being the primary energy storage site, hypodermic fat in endotherms serves a second function, *viz*. insulation (148). No such insulation is required in an ectotherm, which then may explain differential leptin functioning (see below).

In some fish, leptin inhibits food intake through an interaction with hypothalamic orexigenic and anorexigenic genes, as it does in mammals. For example, in goldfish, administration of heterologous (murine) leptin caused a decrease in food intake, with lower doses needed when applied centrally than when applied peripherally to exert the same effect (149). In rainbow trout, recombinant homologous leptin injected intraperitoneally (144) and recombinant human leptin, administered icv, reduces food intake (150).

In common carp, two *leptin* paralogs were characterized initially, designated *leptin-I* and *leptin-II*, which, given their similarity, are probably the result of the tetraploidization following the “recent” cyprinid WGD ~16 Mya (11, 136, 138). These were later renamed *leptin-a* paralogs. The common carp *leptin-a* paralog did not respond to 6 weeks of fasting, nor to feeding to satiation, which feeding regime caused the experimental fish to grow twice as fast as controls. Only transient postprandial increase and decrease were observed in hepatic *leptin-a-II* and *leptin-a-I* mRNA, respectively (136).

Reduction of food intake is an early response of fish when exposed to stress in general (18) and hypoxia in particular (151, 152); in mammals, leptin levels are known to increase in hypoxic conditions (153). Indeed, the mammalian *ob* gene contains hypoxia response element (HRE) sites which can be bound by HIF-1α, a transcription factor that regulates the expression of hypoxia-sensitive genes (153–155). Analyses of the promotor region of zebrafish *leptin* and the *leptin receptor* (*lepr*) revealed putative HRE in both genes (156). Therefore, a follow-up study with common carp was done to elucidate the relation between leptin, hypoxia, and the hypothalamic regulation of food intake. It was shown that hepatic *leptin-a-I, leptin-a-II*, and *lepr* expression in common carp is indeed stimulated in hypoxic conditions, which is congruent with a reduction in food intake (156). This fits with a well-known strategy among fishes to deal with hypoxia (152, 157, 158), since appetite suppression leads to precious energy and oxygen savings by reducing the cost of specific dynamic action (i.e., the metabolic energy cost of digestion) (159). These results were subsequently confirmed by a study in zebrafish, in which chronic hypoxia and HIF-1α induced a rise in hepatic *leptin* mRNA levels (160).

The Bernier laboratory (2012) compared hypothalamic gene expression between hypoxic and fasted (pair-fed to the hypoxic groups) carp, which led to the suggestion that during hypoxia, leptin counteracts the suppression of *pomc* and upregulation of *agrp*, characteristic for fasted carp (136), since *agrp* levels were not affected in hypoxia and the suppression of *pomc* was attenuated (156). These data provide grounds for the involvement of leptin in re-establishing energy balance during chronic hypoxia and indicate a broader physiological role for leptin beyond the signaling of nutrient status.

## Leptin: State of the Art

More than 20 years after the discovery of leptin, a picture emerges of a pleiotropic cytokine, apart from its well-known roles in regulation of appetite and energy balance in mammals, which relates energy status to adaptive responses of multiple physiological systems within vertebrates. In a comprehensive review on mammalian leptin physiology, Friedman (161) elaborates on leptin involvement in the entire neuro-endocrine axis: he points out that already in 1991, Bray noted that *ob/ob* mice are infertile, euthyroid sick, hypothermic, and diabetic (162). In addition, *ob/ob* mice have increased corticosterone levels and immunological and hematological abnormalities. Most of these abnormalities are linked with starvation, not with obesity (163); the lack of leptin signaling to inform the brain that adequate fat stores are present, elicits physiological responses that reduce energy expenditure and stimulate appetite, similar to when the organism is starving (161).

In fish, leptin functions appear to share similarities, but also differences with their mammalian orthologs. Leptin and its receptor have now been cloned for all major vertebrate classes, including the notoriously elusive leptin in birds (164–166). The aim of this section is to provide a concise overview of the functional divergence and evolution of the leptin system, and to gain insights in recently discovered leptin functions in fish.

### Evolution of Leptin and Leptin Receptor Genes

After the initial cloning of leptin in carp, multiple *leptin* paralogs were found in zebrafish (138) and in the Japanese ricefish [*Oryzias latipes*, aka medaka (145)] and named *leptin-a* and *leptin-b*. Indeed, also in common carp a *leptin-b* paralog was found (167) and the first characterized carp leptins were renamed *leptin-a-I* and *leptin-a-II*. The *leptin-a* and *leptin-b* paralogs most likely find their origin in the third, fish-specific, genome duplication, as they are found in evolutionary distant species, such as zebrafish (*Cypriniformes*) and medaka (*Beloniformes*), that shared their last common ancestor ~296 Mya (168).

**Figure 2:**
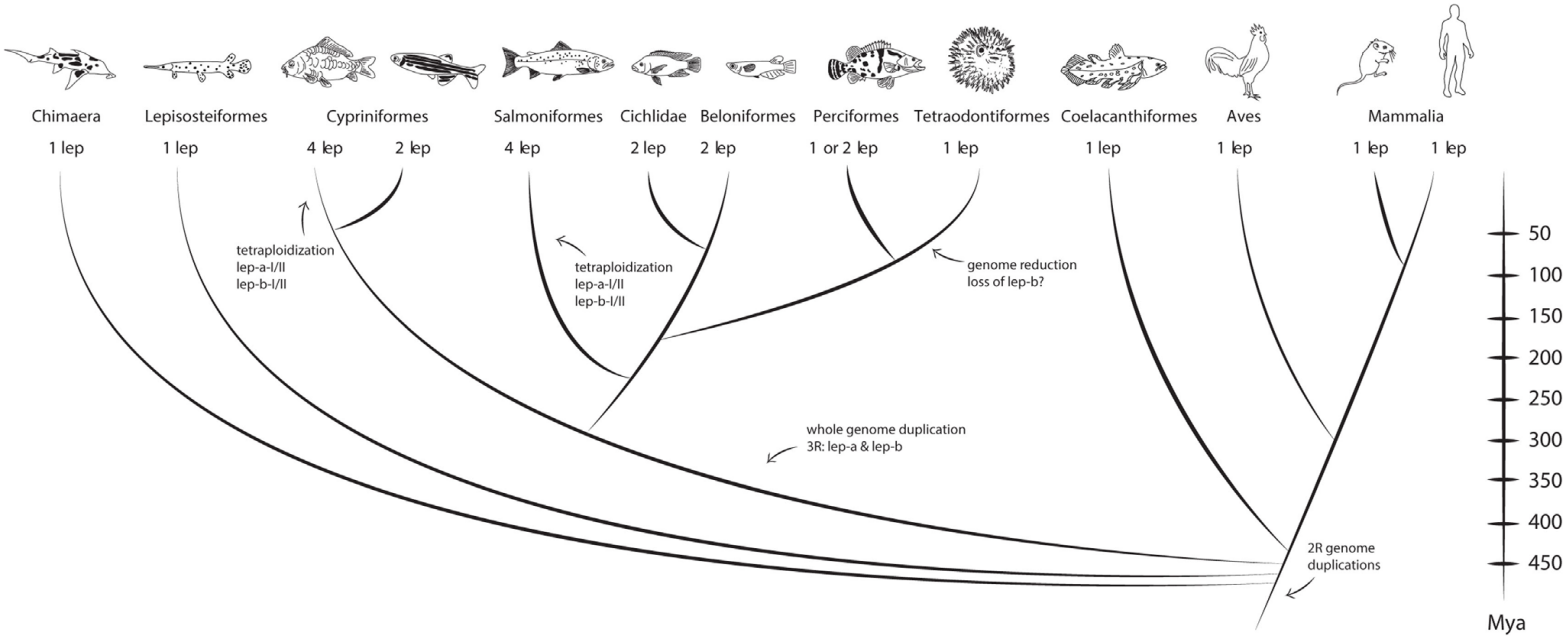
**Vertebrate leptin phylogeny**. Fish and mammals share a common ancestor that lived ~450 Mya. Depending on whole genome duplications one, two, or even four leptin paralogs are found in a species. Recently, the chicken leptin ortholog was found (171). Divergence times come from Near et al. (170). Figure adapted from Gorissen and Flik (165).

However, reflecting the diversity among teleostean fishes, leptin phylogeny appears to be even more complex (Figure 2). Also in the genome of salmonids, four leptin paralogs (*leptin-aI/II* and *leptin-bI/II*) are present. These paralogs originated as a result of the salmonid WGD 88–103 Mya (13, 139, 169). In more modern fish, e.g., the Tiger puffer (Tetraodon family), only a single *leptin* gene is found, which suggests that this lineage experienced genome reduction, after the split from the *Beloniformes* ~186 Mya (170).

As some of the leptin paralogs share very low primary amino acid sequence identity even within a species [e.g., zebrafish leptin-a and leptin-b amino acid identity is only 24% (138)], it is likely that these leptin genes acquired different functionality (neofunctionalization or sub-functionalization) (165). Testimony to this reasoning is that *leptin-a* and *leptin-b* are expressed differentially (at least spatially); *leptin-a* is mainly expressed in the liver (138, 139), while the ovary is the main expression site for *leptin-b* (138). In addition, calculations on binding energy suggest that in both medaka and zebrafish, leptin-a has a higher binding energy for the leptin receptor than leptin-b (172).

Surprisingly, in all currently available teleostean genomes only one *lepr* gene is found (165). In salmonids [Atlantic salmon; Rønnestad et al. (139), and rainbow trout; Gong et al. (173)], several splice variants have been identified, but, as in the mammalian situation, only one of these variants contains the full length sequence required for intracellular signal transduction. In Atlantic salmon, four truncated and one full length leptin receptor variants were identified, the latter of which was ubiquitously expressed, including in gills, gonads, and brain (139), whereas in rainbow trout at least three splice variants gave rise to functional circulating leptin binding proteins (LepBP1, LepBP2, and LepBP3) (173). Together, these findings suggest a complex mode of leptin functioning in teleostean fish, with both inter- and intraspecies differences in the interaction of leptin (paralogs) with the leptin receptor, and in the modulation of endocrine and/or paracrine signaling pathways.

### Recent Discoveries on Leptin Physiology

In the past 5 years, research on leptin functioning in teleostean fish has been carried out not only in the field of feeding and appetite regulation (156, 174–177) but has extended to other endocrine regulatory systems, including sexual maturation (169, 178), energy expenditure and metabolic rate (179, 180), osmotic adaptation (181, 182), and glucose homeostasis (183), all of which we will discuss below. In addition, the involvement of leptin in reproduction (184), the growth hormone (GH)–insulin-like growth factor axis [GH–IGF axis (185)], hypothalamic neurotensin networks (186), and the immune system (187) has been studied.

#### Feeding and Appetite Regulation

The early observations by Huising et al. (136, 137) that *leptin-a* in common carp is regulated by feed intake postprandially are strengthened by a study by Klaren et al. (174). Juvenile common carp were either fed once daily, or demand fed (*via* operating a pendulum connected to an automatic feed dispenser). An interaction effect between time of day and feeding regime was observed for hepatic *leptin-a* transcript abundance (174), indicating an effect of short-term feeding status on *leptin* expression.

Hypoxia-induced elevation of *leptin* mRNA levels, congruent with a reduction in food intake, has been demonstrated in carp (156). MacDonald et al. (176) investigated the effect of hypoxemia on hepatic leptin expression, plasma leptin levels, and food intake in rainbow trout. To establish the hypoxic state, fish were infected with a pathogenic hemoflagellate, *Cryptobia salmositica*, which was followed by an immediate drop in hematocrit. After 14 weeks, infected fish had consumed 75% less food compared to controls, an observation accompanied by an initial, transient, 17-fold increase in hepatic *leptin-a1* expression, and a sustained increase in plasma *leptin-a1* levels. Non-infected fish, pair-fed to the infected group, did not exhibit differences in liver *leptin-a1* expression and plasma leptin compared to non-infected fish fed to satiety. Thus, *leptin-a1* expression appears to be stimulated by hypoxemia, not feed restriction (176), consistent with the results obtained in carp.

Accordingly, no role for leptin in the regulation of food intake in rainbow trout was observed by Jørgensen et al. (177). Plasma leptin levels did not change upon fasting for 4 months, compared to controls, nor after consumption of a large meal. Levels of *leptin-a1* mRNA increased in the belly flap of the fasted fish and stayed high, even after re-feeding, which suggests a tissue-specific role of leptin in long-term fasting. Transcript levels of *pomc* were elevated in fasted fish, possibly to serve as a satiety signal to reduce energy expenditure when food is scarce. However, no causative role in appetite regulation was found for leptin and hypothalamic neuropeptides (177).

To study leptin functioning in medaka, Chisada et al. (175) generated the first fish model deficient of a functional leptin receptor, by inducing a homozygous mutation in the *lepr* gene. Loss of function of the leptin receptor was suggested by disrupted leptin signaling: the expression of appetite-regulating genes differed between mutant and wild-type fish. Independently of feeding, the mutants exhibited constant and upregulated mRNA levels of orexigenic *npya* and *agrp*, but diencephalic *pomc1* expression was downregulated. The post-juvenile and adult mutants displayed hyperphagia, resulting in a high growth rate in the post-juvenile stage, but not in an altered final body size compared to wild-types. In addition, mutants had large deposits of visceral fat, whereas wild-type fish had none (175). These results suggest a stage-specific influence for leptin in food intake, growth, and fat allocation in medaka.

We see that leptin serves different functions with respect to feeding and metabolism among teleosts. Possibly, this is due to differences between species in lipid metabolism and energy storage sites (188).

#### Sexual Maturation

In fish, reproduction is dependent on a healthy energy status. Recently, the focus has been on the role for leptin in sexual maturation in Atlantic salmon. A possible role for leptin in the sexual maturation of male parr (freshwater, FW, stage) has been proposed by Angotzi et al. (169), since they observed higher hepatic *leptin-a1* mRNA levels during mid-spermatogenesis compared to immature fish. However, plasma leptin levels did not differ, so the physiological relevance of these findings is not clear (169).

A seasonal study on the link between leptin and energy balance during sexual maturation of the same species revealed that hepatic *leptin* expression was upregulated during mid-spermatogenesis with a 7.7-fold increase of *leptin-a1* and a 49-fold increase of *leptin-a2* during final maturation (178). For the first time in fish, an upregulation of *lepr* mRNA was observed in the testis from mid- to late spermatogenesis. In non-maturing control fish, the hepatic expression of *leptin-a1* and *leptin-a2* and brain *lepr* was downregulated in early spring, coinciding with the start of growth and fat accumulation. The incidence of sexual maturation was also assessed in a feed-restricted group (fed a low-fat diet at a 50% ration of the control group). This resulted in a 53% decrease in sexual maturation incidence and a major upregulation of both liver *leptins* and pituitary *lepr*. It appears that hepatic *leptin* expression and *lepr* expression in the testis are affected by early sexual maturation in male Atlantic salmon. In addition, the results suggest that leptin does not signal as an adiposity signal in Atlantic salmon, as there is an inverse relationship between fat stores and leptin expression (178). Interestingly, zebrafish lacking either leptin paralog or the leptin receptor show no difference in fecundity compared to wild-type fish (183). Apparently, leptin is involved in sexual maturation, but is not critical for reproductive capability.

#### Energy Expenditure and Metabolic Rate

Leptin affects metabolic rate in zebrafish embryos (179). Translation of *leptin-a* was inhibited by injection of an antisense morpholino oligonucleotide (MO) in the 1–8 cell stage, and oxygen consumption and total acid production were used as indicators of metabolic rate. Morphants consumed significantly less oxygen until 48 h post-fertilization (hpf) followed by a lower acid production, compared to wild-type controls. Co-injection of recombinant zebrafish leptin-a and antisense MO rescued these effects. In addition, a significant decrease in heart rate was seen in morphants, and developmental abnormalities in, *inter alia*, the eyes and the inner ear (189). Taken together, these results suggest that, as in mammals, leptin influences metabolic rate in fishes.

Another study in zebrafish embryos showed that leptin, insulin, and α-MSH, increase energy expenditure dose-dependently (180). An assay to measure metabolic rate was developed based on the reduction of non-fluorescent resazurin by NADH2 to fluorescent resorufin (alamarBlue^®^). To validate the results, a compound known to inhibit hypermetabolic effects of leptin in mice [etomoxir, a carnitine palmitoyl transferase I inhibitor (190)] was tested and shown to block the leptin-induced increase in energy expenditure. These results indicate that leptin’s involvement in the endocrine regulation of energy expenditure is conserved in a teleost (180).

#### SW Adaptation

When fish move between FW and SW, they are challenged by opposite osmotic gradients. Proceeding from a plasma osmolality of 300 mOsmol kg^−1^ this gradient in FW (10 mOsmol kg^−1^; 20°C) results in an osmotic pressure of 706.8 kPa, while in SW (1,000 mOsmol kg^−1^; 20°C) this gradient results in an osmotic pressure of −1,706.1 kPa. Two type-I α-helical cytokines, *viz*. prolactin and GH, play a key role in dealing with these gradients, in FW and SW, respectively. Interestingly, in both cases, cortisol contributes in a synergistic way to hyper- and hypo-osmoregulation (191). Thus, in response to SW exposure, a fish needs to adjust its hydromineral balance, an adaptive and energy demanding process that is associated with enhanced glucose (192) and fatty acid utilization (193). Protein utilization, on the other hand, decreases, as amino acids are retained and function as osmolytes, to maintain cell volume (191, 192). Maybe not surprisingly, considering the energetic cost of osmoregulation, also leptin is involved.

Indeed, in the euryhaline Mozambique tilapia (*Oreochromis mossambicus*), during a 72-h SW challenge, plasma glucose levels were significantly elevated (with a maximum at 12 h after transfer), accompanied by a 25-fold increase in hepatic *leptin-a* expression at 4 h, and elevated *lepr* mRNA levels at 12 h, compared to FW controls (181). To test whether leptin stimulates hepatic glycogenolysis, FW tilapia were injected with recombinant, homologous leptin-a, which resulted in a similar increase in plasma glucose levels as observed during the salinity challenge. Liver glycogen levels were significantly depleted, indicating that leptin-a induced hepatic glycogenolysis necessary for glucose mobilization, to meet increased energy demands during hyperosmotic adaptation (181).

Due to the absence of species-specific antisera, no plasma leptin levels were analyzed in the study described above. In their next study, however, the authors developed and validated an assay to measure plasma leptin-a levels in the Mozambique tilapia (182). This study further identified interactions between prolactin, the pituitary hormone key for adaptation to FW (194, 195), and leptin-a in the euryhaline tilapia. Leptin-a appeared to be the dominant paralog in this species (determined by qPCR analysis of tissues) and is primarily produced by the liver. Hypophysectomized tilapia had higher plasma leptin-a, and hepatic *leptin-a* mRNA levels. These effects could be restored to control values by administration of ovine prolactin. As leptin was found to stimulate *prolactin* expression in the pituitary *in vitro* (196), a negative feedback regulatory model for leptin-a and prolactin seems likely: leptin-a stimulates the expression and secretion of pituitary prolactin (both *prl1* and *prl2*), the prolactins in turn inhibit hepatic *leptin-a* expression, which then translates into a decrease in circulating leptin-a levels (182).

Plasma prolactin and pituitary mRNA levels decrease rapidly upon SW exposure (195). Douros et al. (182) presented tilapia with a 24-h SW challenge, which inactivates the pituitary prolactin cells. During SW acclimation, again a major increase in liver *leptin-a* transcript abundance was observed. Therefore, the authors proposed a mechanism in which the sudden decline in prolactin levels alleviates the continuous inhibition of leptin-a. Prolactin may therefore, *via* leptin-a, be a key glucose regulator in the adaptation to SW (182).

Growth hormone serves to control somatic growth both in FW and SW, and prepares the fish for SW entry by increasing ionoregulatory capacity (197); moreover, GH is particularly well known as permissive for SW adaptation (191). Tilapia leptin-a decreases pituitary *gh* mRNA and hypophysectomy increases *leptin-a* expression, which is rescued by GH replacement. Additionally, during fasting leptin-a enhances hepatic *gh receptor 1* & -*2* and *igf1* & -*2*, to prepare the hepatosomatic growth axis in case feeding resumes (198). We now better understand the GH-IGF axis and its control by leptin-a in the euryhaline tilapia, as leptin, the energy signal, directly steers the endocrine growth axis and, together with cortisol, controls the expensive energy expenditure related to SW adaptation.

Both studies by Baltzegar et al. (181) and Douros et al. (198) on hyperosmotic adaptation in tilapia provide original evidence that leptin-a acts as a potent hyperglycemic factor in tilapia, which is functionally distinct from leptin’s actions in mammals. It is, therefore, an attractive hypothesis that the functional divergence of the leptin protein among vertebrates reflects fundamental differences in metabolic regulation between ectotherms and endotherms (181). The interplay between these three type-I helical cytokines, *viz*. prolactin, GH, and leptin, once again strengthens the epithet *pleiotropic* of this group of hormones [reviewed in Ref. (137)].

#### Glucose Homeostasis

In an elegant series of experiments, Michel et al. (183) demonstrated a role for leptin in glucose homeostasis and disproving a role for leptin as an adipostat in zebrafish. To do so, they created a zebrafish with a dysfunctional leptin receptor. These mutant zebrafish did not exhibit increased adiposity or hyperphagia compared to wild-type controls. In addition, no effect of genotype on length or body mass was found in different life stages and fertility appeared to be normal in these mutants (183).

Given the profound diabetes observed in leptin receptor-deficient mice (*db/db*) (199), and leptin being an important hyperglycemic factor in tilapia (181), it is very tempting to speculate that leptin receptor deficiency in zebrafish would have an effect on glucose homeostasis. Indeed, it seems that, at least in zebrafish, a role for leptin in glucose homeostasis is more pronounced than a role as an adipostat. In the zebrafish leptin receptor mutant, leptin is not required for adipostasis, reproductive functions, or appetite regulation. However, several aspects of glucose homeostasis were altered in mutant fry compared to controls: a small increase in whole body glucose content was found, the expression of the preproinsulin gene *insulin-a* (*insa*), not *insb*, was enhanced in endocrine pancreas tissue, the number of β-cells was 25% higher than in controls, and the expression of key enzymes involved in hepatic glucose metabolism was altered (183). A 3-day exposure of mutant larvae to metformin [a drug known for its beneficial effects on hepatic glucose homeostasis and insulin sensitivity in diabetes patients (200)] normalized the number of β-cells to wild-type levels at 5 days post fertilization. Mutant *leptin-a* and *leptin-b* zebrafish (generated using CRISPR technology) confirmed that lack of leptin-a signaling *via* the leptin receptor is responsible for the increased number of β-cells (183).

With respect to the involvement of leptin in the regulation of food intake and adipostasis, the zebrafish and medaka studies [Michel et al. (183) and Chisada et al. (175), respectively] present opposing results. Whereas Michel et al. (183) concluded that leptin plays a role in glucose homeostasis in zebrafish, but not in adipostasis, Chisada et al. (175) concluded that leptin exerts a powerful influence on food intake regulation and fat allocation in medaka. Although medaka and zebrafish are evolutionary distant species [~296 Mya apart (168)], which could explain differential functions of leptin, the effect of differences in genetic background and raising density in the medaka study cannot be ruled out. A study on knock-out medaka with proper genetic background controls should resolve this issue.

## Leptin and Stress

Re-establishing energy balance is pivotal for vertebrates to realize general homeostasis and cope with environmental or physical disturbances (18, 201, 202). To cope with a (potential) stressful event, vertebrates have to adjust neural, endocrine, and immune mechanisms (203), that, together, modulate energy metabolism. This is an allostatic response; i.e., the ability of an animal to acquire “stability through change” (204, 205). Allostasis is essential in attaining homeostasis (203), and leptin has been annotated as an allostatic hormone (206). This description embraces leptin functioning as a pleiotropic hormone, involved in redistribution of energy, independent of context.

The stress response is largely conserved from fish to terrestrial vertebrates (18, 201), and recently reviewed with respect to fish (202). As both leptin and corticotropin-releasing factor (CRF) are important modulators of energy balance, a link between these hormone systems was predicted soon after the discovery of leptin. Indeed, icv injections of recombinant leptin in fasted rats resulted in increased *Crf* mRNA levels in the hypothalamus, decreased *Npy* expression, and a reduction in food intake (207, 208). Leptin receptors appeared to be concentrated in the ARC (207), thus leptin exerts its anorexic effect, at least in part, mediated by indirect stimulation of CRF *via* the ARC and paraventricular nucleus in mammals (208).

Nutritional state is a crucial component in stress axis activity (209). The contribution of leptin and CRF to the regulation of the stress axis and energy homeostasis is dependent on shared signaling pathways with complementary effects centrally and peripherally. The ultimate result of the stress response is the production and release of glucocorticoids that stimulate the induction of gluconeogenic enzymes in the liver and lipogenesis (210). In mammals, peripheral leptin functions directly at the level of the adrenal gland, where it reduces cortisol release and blunts the adrenal corticotropic hormone (ACTH)-induced rise in cortisol levels (211). Thus, leptin stimulates CRF release from the hypothalamus, but counteracts the peripheral effects of glucocorticoids by inhibiting cortisol release from the adrenal gland.

Also in fish, leptin has been shown to modulate the stress response at multiple levels (156, 212). We have already considered the upregulation of hepatic *leptin* expression during chronic hypoxia in common carp (156), and another study with this species demonstrated that recombinant (human) leptin decreased regulated, CRF-mediated, as well as constitutive ACTH release, and lowered basal cortisol secretion from the head kidney (212). Leptin may then serve as a master signal to downplay the stress response and decrease energy expenditure, as these two processes are intimately linked. This situation is strongly reminiscent of the role of leptin in the GH-IGF axis, as well as the interaction with prolactin.

## Synthesis and Perspectives

Vertebrates have adapted to essentially all niches found on earth, aquatic, terrestrial, and aerial. The conquest of and adaptations to these niches come with niche-specific energetic consequences. The earliest vertebrates evolved in aquatic niches, and their well-lubricated integument is an adaptation to save energy spent on transportation. Swimming is energetically cheaper than flying and running (the most expensive mode of transport). Efficient (aerobic) production of ATP requires a guaranteed oxygen uptake machinery, which is found in high sophistication in the gills of extant fish. The delicate barrier of the gills that facilitates oxygen diffusion comes with a cost: the large branchial surface holds the danger of unwanted water and ion flows, in hypo- or hypersaline waters. Accurate hydromineral balance is secured by the energetically expensive Na^+^/K^+^-ATPase. Fish play with the surface area required for oxygen uptake, but also show metabolic suppression when hypoxic or anoxic conditions arise.

Although several trials with regional endothermy are found in fish, with the transition to land the evolution of true endothermy is seen. The terrestrial environment required more expensive modes of transportation, facilitated by the large metabolic scope inherent to endothermy. Heat loss through air is considerably less than through water. The keratinized skin was equipped externally with feathers or hair and internally with a hypodermic insulating fat layer to retain heat. At the same time, the fat tissue is the major energy depot, which secures the energy requirements of endothermy. In many terrestrial animals, the fat is the largest endocrine tissue, production site, and target of humoral factors key in energy metabolism. Of note, in some icefishes fat can make up 50% of total body mass, fat that serves a role in buoyancy and vertical migration (213).

In the first part of this review, we have non-exhaustively discussed different metabolic strategies. Metabolism, specifically metabolic rate, is at the basis of thermal regulation in both ecto- and endotherms. Metabolism, thermoregulation, and aerobic performance affect each other and all depend on food intake, food being the source of chemical energy. Diametric differences in energy metabolism resulted in different endocrine mechanisms, regulating energy balance and food intake. In the second part of this review, we discussed these endocrine mechanisms in a comparative way, with a focus on insulin and (mainly) leptin.

Up till now, major differences in leptin function between fish species were reported. There is urgent need to find a “common denominator” in teleostean leptin physiology. Most of our current knowledge arises from studies on cyprinids and salmonids, which reflect only a small share of the teleostean diversity. Therefore, one should study leptin in a truly comparative way, including a broader range of fish species. Furthermore, studies on the endocrinology of energy balance should also include insulin. An interesting new avenue of research is the contribution of VAN to energy balance in early vertebrates, and the effects of insulin and leptin thereon.

Insulin and leptin are evolutionary old and the pinnacle regulators of energy intake, storage, and expenditure. Our recent knowledge, in particular on the involvement of leptin in the entire neuro-endocrine axis, is greatly enhanced by comparative studies between early vertebrates and mammals. The evolution of and the interactions within the type-I helical cytokine family (including leptin, prolactin, and GH) elaborated sophisticated control of the energy balance in challenging niches. In addition, comparative studies keep promise to solve the paradoxical (?) evolution of endothermy.

## Author Contributions

IP drafted the manuscript. IP, GF, and MG, all edited and finalized the manuscript.

## Conflict of Interest Statement

The authors declare that this review was written in the absence of commercial or financial relationships that could be interpreted as possible conflicts of interest.

## Footnotes

^1^http://www.engineeringtoolbox.com/oxygen-solubility-water-d_841.html.
