# Supplementary material for: Comparative Physiology of Energy Metabolism: Fishing for Endocrine Signals in the Early Vertebrate Pool
Source: Front Endocrinol (Lausanne). 2017 Mar 2;8:36. doi: 10.3389/fendo.2017.00036 (PMC5332387; doi:10.3389/fendo.2017.00036)
Supplement: Supplementary file 1 [file Data_Sheet_1.PDF]

Glossary of thermal physiology

Bradymetabolism: a metabolic mechanism that implies a slow metabolic rate. Ectothermic vertebrates (most fish, amphibians and reptiles) are bradymetabolic animals.

Ectothermy: the body temperature is governed by environmental and behavioral conditions. Most fish, amphibians, and reptiles rely on external heat to regulate their body temperature.
(Cowles 1940) (Clarke & Pörtner 2010)

Endothermy: a relatively high and constant internal body temperature is maintained. Heat production is governed by a high resting metabolic rate. Of living vertebrates, only birds and mammals exhibit this kind of thermal physiology.
(Cowles 1940) (Clarke & Pörtner 2010)

Heterothermy: a relatively high internal temperature can be, temporarily and spatially, maintained in certain organs or tissues by use of metabolic heat. Within teleostean fishes, the tuna is one of the few species capable of achieving this, which allows for higher energetic output of *e.g.* myotomal muscles.
(Clarke & Pörtner 2010; Graham & Dickson 2001)

Homeothermy: a relatively high and constant internal body temperature is maintained, *via* either an internal or external heat source. This is the case in most endotherms and some large ectotherms, the latter of which have a small surface to volume ratio that leads to a slow loss of metabolic heat, aka *inertial homeothermy* or *gigantothermy*.
(Clarke & Pörtner 2010; Paladino *et al.* 1990)

Poikilothermy: the body temperature is variable. This is the case in many ectothermic animals and many small mammals and birds.
(Clarke & Pörtner 2010)

Tachymetabolism: a metabolic mechanism that implies a fast metabolic rate. Endothermic vertebrates (birds and mammals) are tachymetabolic animals.

Suggested reading:

Clarke A, Pörtner HO. 2010. Temperature, metabolic power and the evolution of endothermy. Biological Reviews 85(4):703-727.
Cossins AR, Bowler K. 1987. Temperature biology of animals. London: Chapman and Hall.
Cowles RB. 1940. Additional implications of reptilian sensitivity to high temperatures. American Naturalist 74:542-561.
Graham JB, Dickson KA. 2001. Anatomical and physiological specializations for endothermy. In: Block BA, Stevens ED, editors. Tuna: physiology, ecology, and evolution. USA: Elsevier Academic Press. p. 121-165.
Paladino FV, O'Connor MP, Spotila JR. 1990. Metabolism of leatherback turtles, gigantothermy, and thermoregulation of dinosaurs. Nature 344(6269):858-860.
